# Manejo das Lesões Circulatórias Sistêmicas Dependentes do Canal no Período Neonatal: Uma Experiência de Dez Anos

**DOI:** 10.36660/abc.20230731

**Published:** 2025-03-18

**Authors:** Dilek Dilli, Hasan Akduman, Rumeysa Çitli, Utku Arman Örun, Vehbi Doğan, Mehmet Taşar, Tamer Yoldaş, Nurdan Dinlen Fettah, Ahmet Özyazıcı, Ayşegül Zenciroğlu

**Affiliations:** 1 University of Health Sciences of Turkey Ankara Dr Sami Ulus Maternity and Children Hospital Department of Neonatology Ankara Turquia University of Health Sciences of Turkey, Ministry of Health of Turkey, Ankara Dr Sami Ulus Maternity and Children Hospital, Department of Neonatology, Ankara – Turquia; 2 University of Health Sciences of Turkey Ankara Dr Sami Ulus Maternity and Children Hospital Department of Pediatric Cardiology Ankara Turquia University of Health Sciences of Turkey, Ministry of Health of Turkey, Ankara Dr Sami Ulus Maternity and Children Hospital, Department of Pediatric Cardiology, Ankara – Turquia; 3 University of Health Sciences of Turkey Ankara Dr Sami Ulus Maternity and Children Hospital Department of Pediatric Cardiovascular Surgery Ankara Turquia University of Health Sciences of Turkey, Ministry of Health of Turkey, Ankara Dr Sami Ulus Maternity and Children Hospital, Department of Pediatric Cardiovascular Surgery, Ankara – Turquia

**Keywords:** Recém-nascido, Coração, Ferimentos e Lesões, Aorta Torácica, Anormalidades Cardiovasculares

## Abstract

**Fundamento:**

Em lesões cardíacas do lado esquerdo dependentes do canal, a circulação sistêmica depende do fluxo da direita para a esquerda através do canal arterial. Essas lesões podem ocorrer como um defeito isolado ou uma doença complexa.

**Objetivo:**

Neste estudo, objetivamos investigar os resultados neonatais de lesões circulatórias sistêmicas dependentes do canal, especialmente coarctação da aorta (CoA) e arco aórtico interrompido (AAI).

**Métodos:**

Um total de 159 pacientes com lesões sistêmicas dependentes de canal foram acompanhados na UTIN Cardíaca de nossa instituição de 2012 a 2022. Revisamos retrospectivamente os prontuários médicos de todos os pacientes do banco de dados do hospital. Eles foram analisados para desfechos clínicos e cirúrgicos. Um valor de p < 0,05 foi considerado estatisticamente significativo.

**Resultados:**

De 159 pacientes, CoA foi detectada em 120 (75,4%) e AAI em 39 (24,5%) pacientes. Cateterismo cardíaco foi realizado em 74 (61,6%) pacientes com CoA no período neonatal; 49 (40,8%) foram submetidos a procedimentos terapêuticos e 25 (20,8%) diagnósticos. Cento e um pacientes com CoA (84,1%) foram submetidos à cirurgia com idade mediana de 14 dias (9-23). Trinta e quatro de 39 pacientes com AAI (87,1%) foram submetidos à cirurgia; reparo em estágio único foi realizado em 13 pacientes (38,2%), enquanto reparo em dois estágios foi aplicado a 21 (61,7%) pacientes. A taxa geral de mortalidade neonatal foi de 19,5% (n=31). Na análise multivariada, as categorias STAT mais altas (OR:2,3, IC:95%, 1,1-5,1, p=0,03) e a presença de complicações pós-operatórias graves (OR:9,8, IC:95%, 2,1-35,1, p=0,003) aumentaram o risco de mortalidade neonatal.

**Conclusão:**

Recém-nascidos com anomalias aórticas congênitas dependentes do canal necessitam de cuidados perioperatórios meticulosos devido ao risco elevado de morbidade e mortalidade.

## Graphical abstract

**Figura Central:**
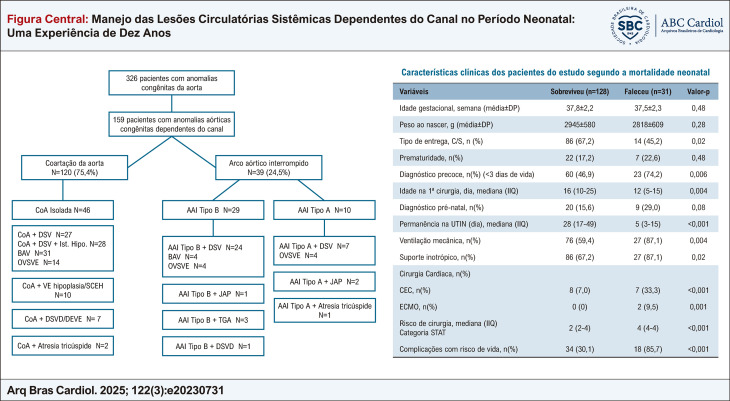
: Manejo das Lesões Circulatórias Sistêmicas Dependentes do Canal no Período Neonatal: Uma Experiência de Dez Anos

**Figura Central:** : Manejo das Lesões Circulatórias Sistêmicas Dependentes do Canal no Período Neonatal: Uma Experiência de Dez Anos

CoA: Coarctação da aorta, Arco aórtico interrompido, SDMO: Síndrome de disfunção de múltiplos órgãos, UTIN: Unidade de terapia intensiva neonatal, CEC: Circulação extracorpórea, ECMO: Oxigenação por membrana extracorpórea, STAT: The Society of Thoracic Surgeons-European Association for Cardio-Thoracic Surgery.

## Introdução

Lesões congênitas dependentes do canal que afetam o lado esquerdo do coração dependem do canal arterial patente (CAP) com um shunt da direita para a esquerda para o fluxo sanguíneo sistêmico. Condições notáveis incluem coarctação da aorta (CoA) e arco aórtico interrompido (AAI), que são proeminentes no período neonatal precoce. Essas lesões exibem um espectro de complexidade, variando de CoA isolada à síndrome do coração esquerdo hipoplásico (SCEH). As abordagens de tratamento estão intrinsecamente ligadas à gravidade da patologia subjacente.^1-6^ CoA refere-se a um estreitamento distinto na aorta, levando à obstrução do fluxo sanguíneo. No AAI, falta continuidade luminal e anatômica entre as partes ascendente e descendente da aorta.^7^ Defeitos do septo ventricular isolados (DSVs) são as lesões associadas mais frequentes (72%) tanto no CoA quanto no AAI. Além disso, podem ser acompanhados por obstrução do trato de saída do ventrículo esquerdo (OVSVE), truncus arteriosus, janela aortopulmonar (JAP), dupla saída do ventrículo direito (DSVD)/dupla entrada do ventrículo esquerdo (DEVE) ou transposição das grandes artérias (TGA).^8^ Estreitamentos aórticos podem ser efetivamente tratados por meio de angioplastia com balão e/ou cirurgia. Dado que o AAI é considerado uma emergência cardíaca, a intervenção cirúrgica imediata é imperativa para reconstruir a aorta e restaurar a função cardíaca normal.^9-11^ Embora o método tradicional de dois estágios^12^ tenha sido historicamente empregado para correção do AAI, a introdução do reparo em estágio único em 1975^13^ ganhou preferência em inúmeras instituições, mesmo para cirurgias complexas, desde que não haja contraindicações para circulação extracorpórea (CEC). Várias modalidades de tratamento para patologias do arco aórtico apresentam vantagens e desvantagens distintas em relação à mortalidade e morbidade.^14-16^

Este estudo apresenta uma análise retrospectiva de dez anos de nossa experiência no tratamento de CoA e AAI, com foco específico nos resultados neonatais.

## Métodos

Este estudo clínico retrospectivo investiga neonatos (com idade <30 dias) apresentando lesões circulatórias sistêmicas dependentes de canal. Os indivíduos foram acompanhados na seção de Unidade de Terapia Intensiva Neonatal Cardíaca (UTIN) do nosso Centro Cardíaco Pediátrico de janeiro de 2012 a agosto de 2022. A aprovação ética para o estudo foi obtida do Comitê de Ética em Pesquisa Clínica Local (E-21/02-090).

**Figura 1:**
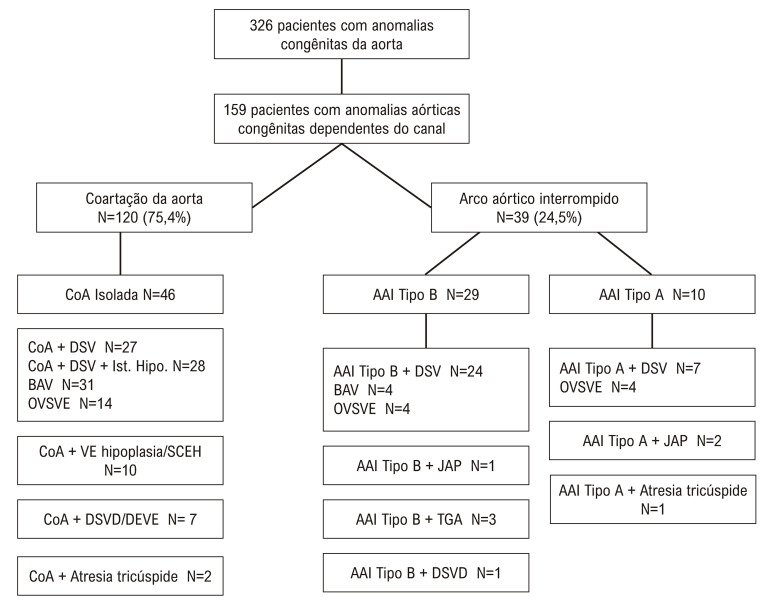
– Tipos de anomalias aórticas congênitas em todos os pacientes do estudo. CoA: coarctação da aorta; AAI: aArco aórtico interrompido; DSV: defeito do septo ventricular; JAP: janela aortopulmonar; DSVD: dupla saída do ventrículo direito; SCEH: síndrome do coração esquerdo hipoplásico; VE: ventrículo esquerdo; OVSVE: obstrução do trato de saída do ventrículo esquerdo; TGA: transposição da grande artéria.

Durante o período do estudo, 917 neonatos consecutivos com defeitos cardíacos congênitos (DCC) foram admitidos em nossa UTIN Cardíaca. Entre eles, 326 foram diagnosticados com anomalias congênitas do arco aórtico. As exclusões compreenderam 75 pacientes que não necessitavam de prostaglandina E1, 74 com SCEH isolada e 18 com estenose ou atresia da valva aórtica isolada. Finalmente, o estudo se concentrou em 159 pacientes com CoA e AAI isolados ou complexos (Figura 1).

Todos os pacientes do estudo receberam cuidados abrangentes em nossa UTIN Cardíaca antes e depois do cateterismo e/ou cirurgia, em estreita colaboração com a equipe de cardiologia pediátrica. Nossos especialistas em cardiologia pediátrica realizaram ecocardiografia transtorácica (ECO) e cateterismos. A infusão intravenosa de prostaglandina E1 (PGE1) foi administrada antes da angioplastia com balão e/ou cirurgia para garantir a perfusão adequada dos órgãos terminais. O conselho cardíaco, composto por um anestesiologista, um cardiologista e um cirurgião cardiovascular, determinou a seleção dos tipos de cateterismo e procedimentos cirúrgicos.

Na CoA, a angioplastia com balão foi estrategicamente utilizada para criar uma janela temporal, permitindo tempo para intervenção cirúrgica subsequente para pacientes que não puderam ser submetidos imediatamente à cirurgia devido à coagulopatia, insuficiência renal aguda, insuficiência hepática ou instabilidade hemodinâmica. No AAI, a angiografia diagnóstica foi predominantemente realizada para avaliar a estrutura anatômica detalhada.

### Coarctação da aorta

A CoA isolada foi identificada em casos caracterizados por um estreitamento discreto da aorta. A CoA foi classificada como “complexa” se associada a outras DCCs, como DSV, DSVD, DEVE ou TGA. Hipoplasia do arco aórtico foi definida por um escore z do diâmetro do arco aórtico menor que -2.^6,17,18^

A intervenção por cateter ou cirurgia foi recomendada para pacientes com CoA quando o gradiente de pressão ultrapassou 20 mmHg. A técnica cirúrgica preferida envolveu anastomose estendida ou ponta a ponta entre o arco aórtico e a aorta torácica descendente (reparo direto da anastomose) via toracotomia. A esternotomia mediana foi reservada para pacientes selecionados com arco transverso estreito. A aortoplastia de retalho foi favorecida apenas em um número limitado de casos, conforme decidido por cirurgiões cardiovasculares.

A recoarctação foi definida por um gradiente maior que 20 mmHg entre as extremidades superiores e inferiores em repouso, com ou sem gradiente de pressão ístmica média maior que 20 mmHg, e a presença de uma cauda diastólica no Doppler pulsado na aorta abdominal, conforme detectado durante o acompanhamento por ECO por cardiologistas pediátricos. A hipertensão paradoxal foi caracterizada por pressão arterial igual ou maior que o percentil 95 para a idade pós-natal.^19^

### Arco aórtico interrompido

O AAI foi definido como uma descontinuidade completa ou um cordão fibroso não patente no arco transverso ou istmo aórtico. As características anatômicas do arco aórtico e a localização da interrupção foram classificadas de acordo com o sistema proposto por Celoria e Patton.^20^ Anomalias complexas associadas, como TGA, JAP, DSVD ou DEVE, foram documentadas. O reparo cirúrgico foi programado após estabilização hemodinâmica.

No reparo de estágio único,^13^ o segmento distal da aorta foi anastomosado com a aorta proximal, e defeitos intracardíacos foram simultaneamente abordados sob CEC. Se a aortoplastia e o reparo de DSV fossem realizados como procedimentos separados, era denominado uma operação de estágio duplo. No primeiro estágio, o reparo do arco, a divisão do CAP e a bandagem pulmonar foram conduzidos para controlar a hipercirculação pulmonar. Após a paliação cirúrgica inicial, uma correção total foi realizada entre três e nove meses de idade. Essa abordagem de reparo em estágios foi favorecida em bebês pequenos ou pacientes hemodinamicamente instáveis, incapazes de tolerar CEC.^12^

### Dados clínicos

O conjunto de dados médicos detalhados abrangeu várias variáveis demográficas e clínicas, incluindo idade gestacional, peso ao nascer, sexo, prematuridade (definida como <37 semanas de idade gestacional), sintomas de apresentação, idade pós-natal no diagnóstico de DCC e a presença de condições médicas concomitantes, como hemorragia intracraniana, insuficiência renal aguda e disfunção tireoidiana.^21^ Dados de acompanhamento sobre a necessidade de ventilação mecânica e a administração de medicamentos inotrópicos foram meticulosamente extraídos de registros clínicos. Outros detalhes incluíram lesões cardíacas associadas, idade no cateterismo e/ou cirurgia, os tipos de cateterismo e reparo cirúrgico, abordagem cirúrgica (toracotomia vs. esternotomia), a utilização de CEC, a necessidade de oxigenação por membrana extracorpórea (ECMO), o escore de risco em cirurgia cardíaca (*The Society of Thoracic Surgeons-European Association for Cardio-Thoracic Surgery; STAT*),^22^ recoarctação após angioplastia ou cirurgia, complicações pós-operatórias precoces (<30 dias de vida), duração da internação na UTIN e a taxa de mortalidade neonatal.

Complicações pós-operatórias graves com risco de vida foram meticulosamente definidas, abrangendo síndrome de baixo débito cardíaco (SBDC; marcada por oligúria, taquicardia, má perfusão ou parada cardíaca necessitando de suporte inotrópico de alta dose), sepse, síndrome de disfunção de múltiplos órgãos (SDMO; envolvendo ≥2 disfunções orgânicas), parada cardíaca súbita, hemorragia pulmonar e arritmia. As características clínicas dos participantes do estudo foram cuidadosamente analisadas com foco na mortalidade neonatal. A sobrevida global foi meticulosamente documentada em prontuários médicos, culminando no final do período do estudo em agosto de 2022.

### Análise estatística

A análise estatística foi conduzida usando o SPSS Statistics para Windows (IBM SPSS Statistics para Windows, versão 24.0. Armonk, NY: IBM Corp). A normalidade da distribuição foi avaliada usando o teste de Kolmogorov-Smirnov. As variáveis quantitativas foram apresentadas como média ± desvio padrão (DP) ou mediana com intervalo interquartil (IIQ), dependendo da normalidade dos dados. Valores de frequência e porcentagem (n, %) foram fornecidos para variáveis qualitativas. Análises comparativas foram realizadas nos dados clínicos de pacientes com CoA e AAI. Além disso, todos os pacientes foram estratificados com base na mortalidade neonatal, e uma análise comparativa de seus dados foi conduzida.

O teste qui-quadrado foi usado para análises de variáveis qualitativas. Para comparar dois grupos independentes, o teste-t de Student foi empregado para variáveis que demonstravam uma distribuição normal, enquanto o teste U de Mann-Whitney foi utilizado para variáveis que não estavam em conformidade com uma distribuição normal. A análise de regressão múltipla foi executada para identificar fatores que afetam a mortalidade. Variáveis significativas em comparações pareadas (modo de parto, idade no diagnóstico, idade na primeira cirurgia, suporte inotrópico pré-operatório, necessidade de ventilação mecânica, categoria STAT, presença de complicações pós-operatórias importantes) foram inseridas no modelo. Cada variável preditora foi avaliada usando a razão de chances (OR) e o intervalo de confiança (IC) de 95%. A significância estatística foi definida como p < 0,05.

## Resultados

Os dados ecocardiográficos detalhados de 159 pacientes do estudo são mostrados na Figura 1 e na Ilustração Central. Alguns pacientes tinham mais de uma lesão.

As características demográficas e clínicas dos pacientes são apresentadas na Tabela 1. A maioria dos pacientes (n=139, 87,4%) foi transferida para nossa UTIN Cardíaca de outras UTINs na Turquia. Um total de 61 pacientes (38,3%) puderam ter um diagnóstico correto de DCC após a alta de UTINs de centros externos.

**Tabela 1:** – As características demográficas e clínicas de todos os pacientes do estudo

Variáveis	N=159	CoA (N=120)	AAI (N=39)	Valor-p
Idade gestacional, semana (média±DP)	37,7±2,2	37,7±2,3	37,8±2,06	0,69
Peso ao nascer, g (média±DP)	2920±586	2939±593	2863±565	0,48
Gênero masculino, n(%)	78 (49,1)	60 (50,0)	18 (46,2)	0,67
Tipo de entrega, C/S, n (%)	100 (62,9)	77 (64,2)	23 (59,0)	0,56
Prematuridade (<37 semanas), n(%)	29 (18,2)	24 (20,0)	5 (12,8)	0,31
Idade no diagnóstico, dia, mediana (IIQ)	3 (1-7)	4 (1-7)	2 (1-5)	0,04
Idade na admissão, dia, mediana (IIQ)	6,0 (3-10)	6 (3-9)	6 (2-12)	0,95
Diagnóstico pré-natal, n(%)	29 (18,2)	19 (15,8)	10 (25,6)	0,16
**No período pré-operatório**				
Ventilação mecânica, n(%)	103 (64,8)	72 (60,0)	31 (79,5)	0,02
Suporte inotrópico, n(%)	113 (71,1)	81 (67,5)	32 (82,1)	0,08
Sepse, n(%)	61 (38,4)	40 (33,3)	21 (53,8)	0,02
Lesão renal aguda, n(%)	34 (21,4)	25 (20,8)	9 (23,1)	0,76
Enterocolite necrosante, n(%)	7 (4,4)	6 (5,0)	1 (2,6)	0,51
**Cateterismo cardíaco, n(%)**				
*1ª angiografia*				
*Diagnóstico*	42 (26,4)	25 (20,8)	17 (43,6)	
*Terapêutico*	51 (32,1)	49 (40,8)	2 (5,1)	0,002
*2ª angiografia*				
*Diagnóstico*	5 (3,1)	2 (1,6)	3 (7,6)	
*Terapêutico*	12 (7,5)	11 (9,1)	1 (2,5)	0,02
**Cirurgia Cardíaca, n(%)**				
**1ª Cirurgia**	**135 (84,9)**	101 (84,1)	34 (87,1)	
***Toracotomia***	112 (83,0)	96 (95,0)	16 (47,1)	
***Esternotomia***	23 (17,0)	5 (5,0)	18 (52,9)	<0,001
*CEC*	15 (11,1)	3 (3,0)	12 (35,3)	<0,001
*ECMO*	2 (1,5)	0 (0)	2 (5,9)	0,14
**2ª Cirurgia**	**36 (22,6)**	23 (19,1)	13 (33,3)	
***Toracotomia***	17 (47,2)	13 (56,5)	4 (30,8)	
***Esternotomia***	19 (52,8)	10 (43,5)	9 (69,2)	0,13
*CEC*	14 (38,9)	6 (26,1)	8 (61,5)	0,03
*ECMO*	4 (11,1)	1 (4,3)	3 (823,1)	0,08
Complicações com risco de vida, n(%)	52 (38,8)	29 (29,0)	23 (67,6)	<0,001
Permanência na UTIN, mediana de dias (IIQ)	24 (13-43)	23 (13-43)	24 (13-57)	0,09
Tempo de acompanhamento, mediana (ano) (IIQ)	1 ano (2 meses-6 anos)	3 anos (2 meses-7 anos)	2 meses (1 mês-2 anos)	0,001
Mortalidade neonatal, n(%)	**31 (19,5)**	16 (13,3)	15 (38,5)	0,001

CEC: Circulação extracorpórea; ECMO: Oxigenação por membrana extracorpórea; UTIN: Unidade de terapia intensiva neonatal.

Quinze (9,4%) pacientes eram imigrantes (sírios ou iraquianos). A taxa de parto prematuro foi de 18,2% (n=29). No período pré-operatório, todos os pacientes receberam infusão de PGE1. Vinte pacientes apresentaram aparência sindrômica: síndrome de DiGeorge (n=6), síndrome de Down (n=5), síndrome de Turner (n=2) e trissomia 18 (Edwards) (n=1). O histórico médico materno foi significativo para diabetes gestacional (n=3) e hipotireoidismo (n=2). Terapia hormonal foi necessária para hipotireoidismo em seis pacientes.

No período neonatal, o cateterismo cardíaco foi realizado em 93 dos 159 pacientes (58,4%).

Trombose (n=16, 17,2%), hipotermia (n=9, 9,6%) e sangramento (n=4, 4,3%) foram as complicações mais comuns observadas após cateterismo. De 135 pacientes submetidos à cirurgia, complicações sérias com risco de vida foram observadas em 38,5% (n=52).

A duração média da internação na UTIN foi de 24 dias (13-43). As mortalidades neonatais (19,5%, n=31) foram atribuídas a fatores distintos, abrangendo insuficiência cardíaca pré-operatória e sepse (n=13), parada intraoperatória (n=8) e complicações pós-operatórias como sepse, pneumonia, SDMO e/ou instabilidade hemodinâmica (n=10).

### Coarctação da Aorta

Durante o período neonatal, com idade mediana de 11 dias (6-28), 74 (61,6%) de 120 pacientes com CoA foram submetidos a cateterismo cardíaco pela artéria e/ou veia femoral. Entre eles, 49 (66,2%) foram submetidos a cateterismos terapêuticos (45 angioplastias com balão, quatro valvuloplastias com balão), enquanto 25 (33,7%) foram submetidos a cateterismos diagnósticos (ver Tabela 1). O gradiente de pressão mostrou uma diminuição significativa após a angioplastia [30 (20-40) mmHg vs. 10 (5-14) mmHg, p<0,001)].

Após a angioplastia inicial, 28 pacientes foram submetidos à cirurgia, e três necessitaram de uma segunda angioplastia com balão devido à recoarctação, resultando em uma taxa de recoarctação de 63,2% (n=31/49). Após a angioplastia, hipertensão paradoxal que exigiu tratamento foi observada em oito de 45 pacientes (16,3%).

Em uma idade média de 14 dias (9,2-23 dias), 101 pacientes com CoA (84,1%) foram submetidos à cirurgia (96-toracotomia e 5-esternotomia). Dezenove de 120 pacientes (15,8%) não foram submetidos à cirurgia; onze receberam angioplastia com balão e oito faleceram antes da cirurgia.

Entre 101 pacientes submetidos à cirurgia, anastomose término-terminal de ressecção estendida (n=66, 65,3%), anastomose término-terminal de ressecção (n=19, 18,8%) e reparo de enxerto (n=16, 15,8%) foram os procedimentos cirúrgicos. Três pacientes (2,9%) foram operados sob CEC e parada circulatória hipotérmica moderada. Após a cirurgia, hipertensão paradoxal se desenvolveu em 18 de 101 (17,8%) pacientes. As outras complicações frequentes foram sepse, SDMO, insuficiência renal aguda, quilotórax e paralisia do diafragma. Três pacientes morreram durante a cirurgia e cinco pacientes morreram devido a parada cardíaca súbita pós-operatória ou SDMO.

Com idade média de 88 dias (48-350), 13 pacientes com CoA foram submetidos ao segundo cateterismo; sete foram submetidos à angioplastia com balão, dois cateterismos diagnósticos, uma septostomia atrial e um fechamento de CIV com dispositivo Amplatzer Duct Occluder II (ADOII).

Na infância, 23 pacientes foram submetidos à segunda cirurgia com uma taxa de recoarctação de 22,7% em uma idade média de 148 dias (68-330). Nesse ponto, as cirurgias foram realizadas por toracotomia em 13 (56,5%) pacientes e esternotomia mediana em 10 (43,5%) pacientes. Os tipos de cirurgias foram anastomose término-terminal de ressecção estendida (n=16), reparo de enxerto (n=2), patch de DSV (n=2) e ressecção de OVSVE (n=3). Seis dos pacientes foram submetidos à CEC.

### Interrupção do arco aórtico

De 39 pacientes, cateterismos diagnósticos foram realizados em 17 (43,6%) com idade mediana de 7,5 dias (3-15). Cinco pacientes com AAI (12,8%) não puderam ser submetidos à cirurgia porque faleceram antes da cirurgia. Assim, 34 pacientes (87,1%) foram submetidos ao reparo de AAI com idade mediana de 14 dias (5-20) no período neonatal.

Dos 34 pacientes, 16 (47,1%) foram operados com toracotomia e 18 (52,9%) com esternotomia; 12 (35,3%) foram submetidos à CEC. Foi realizado reparo em estágio único em 12 pacientes: dois reparos de JAP e arco aórtico com enxerto, nove anastomoses término-terminais e reparo de patch de DSV. O reparo em dois estágios foi implementado em 22 pacientes: 10 foram submetidos ao reparo do arco aórtico com enxerto e bandagem pulmonar, nove foram submetidos à anastomose término-terminal com bandagem pulmonar e três foram submetidos à anastomose término-terminal sem bandagem pulmonar. No período pós-operatório imediato, dois pacientes (5,9%) necessitaram de ECMO. Os tipos de reparo e as taxas medianas de esternotomia foram semelhantes entre os pacientes com AAI tipo A e AAI tipo B (p > 0,05 para ambas as comparações). Na infância, em uma mediana de 150 dias (45-202), três pacientes necessitaram de cateterismo diagnóstico, e um foi submetido a cateterismo terapêutico (stent endovascular para estenose para-anastomótica).

Um total de 13 pacientes foram submetidos à segunda cirurgia com idade média de 177 dias (56-330). Nesta etapa, as cirurgias foram realizadas por toracotomia em quatro pacientes e esternotomia em nove pacientes. Os tipos de cirurgias foram seis reparos de patch de DSV, quatro trocas de enxerto e três re-bandagens pulmonares. Oito dos pacientes entraram em CEC, enquanto três pacientes precisaram de ECMO.

As complicações pós-operatórias mais comuns foram SBDC, sepse/SDMO, pneumonia, lesão renal aguda e arritmia. SBDC foi definida em cinco pacientes com AAI e está intimamente relacionada à disfunção ventricular esquerda pré-operatória. Dois pacientes faleceram durante a cirurgia, e oito morreram devido a SBDC ou SDMO pós-operatórias. A taxa de mortalidade foi menor pelo reparo em dois estágios, embora não significativamente (50,0% vs. 34,8%, respectivamente, p=0,38).

### Desfechos

A Figura Central e a Tabela 2 mostram as características clínicas dos pacientes do estudo de acordo com a mortalidade neonatal.

**Tabela 2:** – Características clínicas dos pacientes do estudo segundo a mortalidade neonatal

Variáveis	Sobreviveu (n=128)	Faleceu (n=31)	Valor-p
Idade gestacional, semana (média±DP)	37,8±2,2	37,5±2,3	0,48
Peso ao nascer, g (média±DP)	2945±580	2818±609	0,28
Gênero masculino, n(%)	64 (50,0)	14 (45,2)	0,62
Tipo de entrega, C/S, n (%)	86 (67,2)	14 (45,2)	0,02
Prematuridade, n(%)	22 (17,2)	7 (22,6)	0,48
Diagnóstico precoce, n(%) (<3 dias de vida)	60 (46,9)	23 (74,2)	0,006
Idade na 1ª cirurgia, dia, mediana (IIQ)	16 (10-25)	12 (5-15)	0,004
Diagnóstico pré-natal, n(%)	20 (15,6)	9 (29,0)	0,08
Permanência na UTIN (dia), mediana (IIQ)	28 (17-49)	5 (3-15)	<0,001
Ventilação mecânica, n(%)	76 (59,4)	27 (87,1)	0,004
Suporte inotrópico, n(%)	86 (67,2)	27 (87,1)	0,02
Sepse, n(%)	51 (39,8)	10 (32,3)	0,43
SDMO, n(%)	23 (18,0)	11 (35,5)	0,03
Lesão renal aguda, n(%)	25 (19,5)	9 (29,0)	0,24
Diálise peritoneal, n(%)	6 (4,7)	3 (9,7)	0,28
Hemorragia intracraniana/alterações hipóxico-isquêmicas, n(%)	19 (14,8)	3 (9,7)	0,45
Cirurgia Cardíaca, n(%)			
CEC, n(%)	8 (7,0)	7 (33,3)	<0,001
ECMO, n(%)	0 (0)	2 (9,5)	0,001
Risco de cirurgia, mediana (IIQ) Categoria STAT	2 (2-4)	4 (4-4)	<0,001
Complicações com risco de vida, n(%)	34 (30,1)	18 (85,7)	<0,001

CoA: Coarctação da aorta, Arco aórtico interrompido; SDMO: Síndrome de disfunção de múltiplos órgãos; UTIN: Unidade de terapia intensiva neonatal; CEC: Circulação extracorpórea; ECMO: Oxigenação por membrana extracorpórea; STAT: The Society of Thoracic Surgeons-European Association for Cardio-Thoracic Surgery.

Na análise multivariada, as categorias STAT mais altas (OR:2,3, IC:95%, 1,1-5,1, p=0,03) e a presença de complicações pós-operatórias maiores (OR:9,8, IC:95%, 2,1-35,1, p=0,003) aumentaram o risco de mortalidade neonatal. As outras variáveis não afetaram o risco de mortalidade.

O tempo mediano de acompanhamento de todos os pacientes foi de um ano (2 meses-6 anos). A sobrevida global no final do período do estudo foi de 71,0% (n=113/159).

## Discussão

As apresentações clínicas da CoA variam amplamente, abrangendo bebês que apresentam insuficiência cardíaca a indivíduos assintomáticos que apresentam hipertensão sistêmica ou sopros detectados incidentalmente durante exames físicos de rotina em crianças e adultos.^10,23^ A CoA também pode estar associada a síndromes genéticas específicas, incluindo Síndrome de Down, Turner ou Williams-Beuren.^24,25^ Em nosso estudo, os sintomas de apresentação mais prevalentes foram sopros cardíacos, cianose, problemas respiratórios e comprometimento hemodinâmico. Alguns pacientes com CoA foram associados às síndromes de Down, Edwards e Turner.

O tratamento de lesões complexas envolvendo hipoplasia do arco pode ser feito por meio de toracotomia com anastomose término-terminal estendida, visando aumentar o crescimento do arco proximal pela eliminação da obstrução distal.^26^ Um método alternativo envolve esternotomia mediana e alargamento do arco aórtico para obter alívio ideal de qualquer obstrução ema saída do ventrículo esquerdo.^27^ Dharmapuram et al.^28^ relataram resultados positivos usando anastomose término-terminal estendida por meio de toracotomia com técnica cirúrgica modificada em casos de CoA associados à hipoplasia do arco proximal. Gropler et al.^29^ sugeriram que a esternotomia mediana deve ser considerada para pacientes com hipoplasia grave do arco. A aortoplastia com enxerto não é recomendada na infância devido ao risco associado de dilatação aneurismática.^30^

Em nosso estudo, um total de 101 pacientes com CoA foram submetidos à cirurgia, e a maioria dos casos foi tratada com toracotomia. O procedimento preferido para a maioria dos pacientes com CoA foi a ressecção com anastomose término-terminal estendida. Aproximadamente um terço dos pacientes com CoA estão associados à hipoplasia do arco aórtico. Para este subgrupo, a abordagem cirúrgica escolhida envolveu esternotomia com anastomose término-terminal estendida ou a aplicação de remendos aórticos. O reparo do enxerto foi realizado seletivamente em pacientes com hipoplasia de segmento longo. Notavelmente, nossa equipe não empregou esta técnica cirúrgica específica para reparo de CoA em bebês nos últimos cinco anos.

Resultados de médio a longo prazo destacam que a obstrução recorrente do arco aórtico no pós-operatório continua sendo uma preocupação significativa. No estudo de Onalan et al.,^31^ foi descoberto que 20,8% dos pacientes necessitaram de reintervenção devido à recoarctação distal, com sete tratados com sucesso por angioplastia aórtica com balão e três necessitando de reintervenção cirúrgica.

Após o reparo da CoA, a persistência ou recorrência da hipertensão pode estar relacionada a fatores como idade mais avançada no reparo, presença de obstrução residual, formato do arco aórtico subótimo e mecanismos neuro-humorais pouco claros iniciados antes do reparo. A incidência relatada de hipertensão após o reparo da CoA varia entre 17 e 48% em vários estudos.^29-33^ Em nosso estudo, a taxa de hipertensão após a cirurgia de coarctação foi de 17,8%.

Atualmente, a angioplastia com balão é a abordagem preferida para casos selecionados de CoA. No período neonatal, a angioplastia pode ser usada como primeira opção para ganhar tempo até a cirurgia, especialmente em casos de baixo peso ao nascer e instabilidade hemodinâmica. Em nosso estudo, aproximadamente 40% dos casos com CoA foram submetidos à angioplastia. Em um estudo de Oswal et al. envolvendo 44 bebês, o gradiente médio diminuiu de 48,05±15,26 mmHg para 10,97±5,8 mmHg, demonstrando resultados imediatos bem-sucedidos após angioplastia com balão para CoA.^34^No presente estudo, todos os lactentes apresentaram um gradiente de pico de mais de 20 mmHg antes da intervenção. Após a angioplastia, houve uma redução significativa no gradiente de pressão [30 (20-40) mmHg vs. 10 (5-14) mmHg].

Um AAI é um DCC raro e grave, que frequentemente coexiste com outros defeitos intracardíacos e extracardíacos.^8^ Em nosso estudo, oito dos pacientes com AAI tinham defeitos cardíacos complexos associados, como JAP, TGA, DSVD e atresia tricúspide. As taxas de mortalidade foram semelhantes entre os tipos de AAI complexos e isolados. Lidar com malformações complexas apresenta desafios cirúrgicos, exigindo procedimentos intrincados. Em tais casos, a paliação pode ser ineficaz, e o reparo completo precoce é frequentemente necessário após o nascimento.

Nos últimos anos, tem havido uma tendência global em promover o reparo em estágio único para AAI, mas isso requer habilidades técnicas complexas e cooperação multidisciplinar.^12,13^ Nosso estudo envolvendo 39 pacientes com AAI mostrou que 12 foram submetidos a reparo em estágio único, enquanto 22 necessitaram de reparo em estágio duplo.

No período neonatal, complicações pós-operatórias sérias como SBDC podem ocorrer após o reparo do arco aórtico, particularmente quando associadas à disfunção ventricular esquerda pré-operatória. Um estudo de Lim et al.^35^ relatou uma taxa de 5,7% de SBDC pós-operatória em neonatos ou bebês submetidos a reparo total de estágio único de anomalias do arco aórtico com condições pré-operatórias precárias. Nosso estudo também observou SBDC em cinco pacientes com AAI após a cirurgia, todos com disfunção ventricular esquerda pré-operatória. A presença de sepse pré-operatória pode ter impactado negativamente seus resultados.

Embora tanto CoA quanto AAI sejam patologias do arco aórtico,^1,2^ o prognóstico para AAI é mais grave.^7^ Ao comparar os dois grupos de pacientes, observou-se que os casos de AAI foram diagnosticados mais cedo do que os casos de CoA, possivelmente devido aos sintomas mais proeminentes. Da mesma forma, a necessidade de ventilação mecânica, a taxa de sepse e a frequência de IRA foram maiores nos casos de AAI. Na presença de CoA isolada, a angioplastia pode fornecer uma solução temporária, enquanto no AAI, a angiografia é usada para fins diagnósticos, com o tratamento sendo exclusivamente cirúrgico. Portanto, em nosso estudo, a frequência de angiografia terapêutica foi maior nos casos de CoA, enquanto a angiografia diagnóstica foi mais frequente nos casos de AAI.

Embora cirurgias para CoA isolada possam ser realizadas usando métodos minimamente invasivos e toracotomia, procedimentos de AAI são frequentemente realizados sob CEC e por esternotomia. Consequentemente, a frequência de esternotomia foi maior entre os casos de AAI. Além disso, como relatado anteriormente,^8^ devido à complexidade da cirurgia, a categoria STAT, a necessidade de ECMO e as taxas de mortalidade foram maiores em casos de AAI.

Ao comparar casos sobreviventes e falecidos, observou-se que aqueles que morreram receberam um diagnóstico mais precoce. Esse fenômeno foi atribuído à gravidade da doença, levando à manifestação precoce dos sintomas em bebês. Além disso, devido à intervenção cirúrgica mais precoce necessária para casos mais graves, a idade na cirurgia para pacientes falecidos foi menor, e suas categorias STAT foram maiores. Comparados aos sobreviventes, os pacientes falecidos tiveram maior necessidade de ventilação mecânica pré-operatória e suporte inotrópico, e a taxa de SDMO foi mais frequente. No grupo falecido, os requisitos para CEC e ECMO, bem como a frequência de complicações pós-operatórias com risco de vida, foram maiores. Na análise de regressão multivariada, os fatores que mais impactaram significativamente a mortalidade foram a categoria STAT, que reflete a complexidade da cirurgia e o desenvolvimento de complicações pós-operatórias graves.

Este estudo tem limitações, incluindo seu desenho retrospectivo e de centro único, dificultando a generalização dos resultados. Estudos multicêntricos com resultados de longo prazo são necessários para uma compreensão abrangente das anomalias congênitas do arco aórtico.

Concluindo, recém-nascidos com anomalias aórticas congênitas dependentes do canal requerem cuidados perioperatórios ideais devido ao seu risco aumentado de morbidade e mortalidade. Em países em desenvolvimento, melhorar os resultados de DCC neonatal parece possível por meio de vários fatores, incluindo maior conhecimento e experiência dos médicos, a curva de aprendizado alcançada, adaptação aos desenvolvimentos médicos e técnicos e manter os avanços na cirurgia cardíaca neonatal.
