# Supplementary material for: Manejo das Lesões Circulatórias Sistêmicas Dependentes do Canal no Período Neonatal: Uma Experiência de Dez Anos
Source: Arq Bras Cardiol. 2025 Mar 18;122(3):e20230731. [Article in Portuguese] doi: 10.36660/abc.20230731 (PMC12013741; doi:10.36660/abc.20230731)
Supplement: Translation

# Translation: Management of Ductus-Dependent Systemic Circulatory Lesions in the Neonatal Period: A Ten-Year Experience

**DOI:** 10.36660/abc.20230731i

**Authors:** Dilek Dilli, Hasan Akduman, Rumeysa Çitli, Utku Arman Örun, Vehbi Doğan, Mehmet Taşar, Tamer Yoldaş, Nurdan Dinlen Fettah, Ahmet Özyazıcı, Ayşegül Zenciroğlu

**Affiliations:** 1 Türkiye University of Health Sciences of Turkey, Ministry of Health of Turkey, Ankara Dr Sami Ulus Maternity and Children Hospital, Department of Neonatology, Ankara – Türkiye; 2 Türkiye University of Health Sciences of Turkey, Ministry of Health of Turkey, Ankara Dr Sami Ulus Maternity and Children Hospital, Department of Pediatric Cardiology, Ankara – Türkiye; 3 Türkiye University of Health Sciences of Turkey, Ministry of Health of Turkey, Ankara Dr Sami Ulus Maternity and Children Hospital, Department of Pediatric Cardiovascular Surgery, Ankara – Türkiye

**Keywords:** Newborn, Heart, Wounds and Injuries, Thoracic Aorta, Cardiovascular Abnormalities

## Abstract

**Background:**

In ductus-dependent left-sided heart lesions, systemic circulation depends on right-to-left flow via the ductus arteriosus. These lesions may occur as an isolated defect or complex disease.

**Objective:**

In this study, we aimed to investigate the neonatal outcomes of duct-dependent systemic circulatory lesions, especially aortic coarctation (CoA) and interrupted aortic arch (IAA).

**Methods:**

A total of 159 patients with duct-dependent systemic lesions were followed up in the Cardiac NICU of our institution from 2012 to 2022. We retrospectively reviewed the medical charts of all patients from the hospital database. They were analyzed for clinical and surgical outcomes. A p-value of <0.05 was considered statistically significant.

**Results:**

Of 159 patients, CoA was detected in 120 (75.4%) and IAA in 39 (24.5%) patients. Cardiac catheterization was performed in 74 (61.6%) patients with CoA in the neonatal period; 49 (40.8%) underwent therapeutic and 25 (20.8%) diagnostic procedures. One hundred one patients with CoA (84.1%) underwent surgery at a median age of 14 days (9-23). Thirty-four of 39 patients with IAA (87.1%) underwent surgery; single-stage repair was performed on 13 patients (38.2%), while two-stage repair was applied to 21 (61.7%) patients. The overall neonatal mortality rate was 19.5% (n=31). In multivariate analysis, the higher STAT categories (OR:2.3, CI:95%, 1.1-5.1, p=0.03) and the presence of major postoperative complications (OR:9.8, CI:95%, 2.1-35.1, p=0.003) have increased the risk of neonatal mortality.

**Conclusion:**

Newborns with congenital duct-dependent aortic anomalies necessitate meticulous perioperative care due to their heightened risk of morbidity and mortality.

## Introduction

Ductal-dependent congenital lesions affecting the left side of the heart rely on the patent ductus arteriosus (PDA) with a right-to-left shunt for systemic blood flow. Notable conditions include coarctation of the aorta (CoA) and interrupted aortic arch (IAA), which are prominent in the early neonatal period. These lesions exhibit a spectrum of complexity, ranging from isolated CoA to hypoplastic left heart syndrome (HLHS). Treatment approaches are intricately linked to the severity of the underlying pathology.^1-6^ CoA refers to a distinct narrowing in the aorta, leading to obstruction in the blood flow. In IAA, luminal and anatomical continuity is lacking between the ascending and descending parts of the aorta.^7^ Isolated ventricular septal defects (VSDs) are the most frequent associated lesions (72%) in both CoA and IAA. Additionally, they may be accompanied by left ventricular outflow tract obstruction (LVOTO), truncus arteriosus, aortopulmonary window (APW), double-outlet right ventricle (DORV)/double-inlet left ventricle (DILV), or transposition of the great arteries (TGA).^8^Aortic narrowings can be effectively addressed through balloon angioplasty and/or surgery. Given that IAA is considered a cardiac emergency, immediate surgical intervention is imperative for reconstructing the aorta and restoring normal heart function.^9-11^ While the traditional double-stage method^12^ has historically been employed for IAA correction, the introduction of single-stage repair in 1975^13^ has gained preference in numerous institutions, even for complex surgeries, provided there are no contraindications to cardiopulmonary bypass (CPB). Various treatment modalities for aortic arch pathologies exhibit distinct advantages and disadvantages concerning mortality and morbidity.^14-16^

This study presents a ten-year retrospective analysis of our experience in managing CoA and IAA, specifically focusing on neonatal outcomes.

## Methods

This retrospective clinical study investigates neonates (aged <30 days) presenting with duct-dependent systemic circulatory lesions. The subjects were followed up at the Cardiac Neonatal Intensive Care Unit (NICU) section of our Pediatric Heart Center from January 2012 to August 2022. Ethical approval for the study was obtained from the Local Clinical Research Ethics Committee (E-21/02-090).

Over the study period, 917 consecutive neonates with congenital heart defects (CHD) were admitted to our Cardiac NICU. Among them, 326 were diagnosed with congenital aortic arch anomalies. Exclusions comprised 75 patients not requiring prostaglandin E1, 74 with isolated HLHS, and 18 with isolated aortic valve stenosis or atresia. Finally, the study focused on 159 patients with isolated or complex CoA and IAA (Figure 1).

**Figure 1:** – Types of congenital aortic anomalies in all study patients. CoA: coarctation of aorta; IAA: interrupted aortic arch; VSD: ventricular septal defect; APW: aortopulmonary window; DORV: double outlet right ventricle; HLHS: hypoplastic left ventricle; LV: left ventricle; LVOTO: left ventricle outflow tract obstruction; TGA: transposition of the great arteria.

All study patients received comprehensive care at our Cardiac NICU before and after catheterization and/or surgery, in close collaboration with the pediatric cardiology team. Our pediatric cardiology specialists performed transthoracic echocardiography (ECHO) and catheterizations. Intravenous prostaglandin E1 (PGE1) infusion was administered prior to balloon angioplasty and/or surgery to ensure adequate perfusion of end organs. The cardiac council, consisting of an anesthesiologist, cardiologist, and cardiovascular surgeon, determined the selection of the types of catheterization and surgical procedures.

In CoA, balloon angioplasty was strategically utilized to create a temporal window, allowing time for subsequent surgical intervention for patients who could not immediately undergo surgery due to coagulopathy, acute renal failure, liver failure, or hemodynamic instability. In IAA, diagnostic angiography was predominantly performed to assess the detailed anatomical structure.

### Coarctation of the aorta

Isolated CoA was identified in cases characterized by a discrete narrowing of the aorta. CoA was classified as “complex” if associated with other CHDs such as VSD, DORV, DILV, or TGA. Aortic arch hypoplasia was defined by a z score of the aortic arch diameter less than -2.^6,17,18^

Catheter intervention or surgery was recommended for patients with CoA when the pressure gradient surpassed 20 mmHg. The preferred surgical technique involved extended or end-to-end anastomosis between the aortic arch and descending thoracic aorta (direct anastomosis repair) via thoracotomy. Midline sternotomy was reserved for selected patients with a narrow transverse arch. Patch aortoplasty was only favored in a limited number of cases as decided by cardiovascular surgeons.

Recoarctation was defined by a gradient greater than 20 mmHg between the upper and lower extremities at rest, with or without a mean isthmic pressure gradient greater than 20 mmHg, and the presence of a diastolic tail in pulsed-wave Doppler in the abdominal aorta, as detected during ECHO follow-up by pediatric cardiologists. Paradoxical hypertension was characterized by blood pressure equal to or greater than the 95th percentile for postnatal age.^19^

### Interrupted aortic arch

IAA was defined as either a complete discontinuity or a nonpatent fibrous strand in the transverse arch or aortic isthmus. The anatomic features of the aortic arch and the location of the interruption were classified according to the system proposed by Celoria and Patton.^20^ Associated complex anomalies, such as TGA, APW, DORV, or DILV, were documented. Surgical repair was scheduled following hemodynamic stabilization.

In single-stage repair,^13^ the distal segment of the aorta was anastomosed with the proximal aorta, and intracardiac defects were concurrently addressed under CPB. If aortoplasty and VSD repair were performed as separate procedures, it was termed a double-stage operation. In the first stage, arch repair, PDA division, and pulmonary banding were conducted to control pulmonary overcirculation. Following initial surgical palliation, a total correction was carried out between three to nine months of age. This staged repair approach was favored in small infants or hemodynamically unstable patients unable to tolerate CPB.^12^

### Clinical data

The detailed medical dataset encompassed various demographic and clinical variables, including gestational age, birth weight, sex, prematurity (defined as <37 weeks of gestational age), presenting symptoms, postnatal age at the diagnosis of CHD, and the presence of concurrent medical conditions such as intracranial hemorrhage, acute renal failure, and thyroid dysfunction.^21^ Follow-up data regarding the requirement for mechanical ventilation and the administration of inotropic medications were meticulously extracted from clinical records. Further details included associated heart lesions, age at catheterization and/or surgery, the types of catheterization and surgical repair, surgical approach (thoracotomy vs. sternotomy), the utilization of CPB, the need for extracorporeal membrane oxygenation (ECMO), the risk score in cardiac surgery (The Society of Thoracic Surgeons-European Association for Cardio-Thoracic Surgery; STAT),^22^ recoarctation after angioplasty or surgery, early postoperative complications (<30 days of life), length of NICU stay, and the rate of neonatal mortality.

Life-threatening serious postoperative complications were meticulously defined, encompassing low cardiac output syndrome (LCOS; marked by oliguria, tachycardia, poor perfusion, or cardiac arrest necessitating high-dose inotropic support), sepsis, multi-organ dysfunction syndrome (MODS; involving ≥2 organ dysfunctions), sudden cardiac arrest, pulmonary hemorrhage, and arrhythmia. The clinical characteristics of the study participants were thoroughly analyzed with a focus on neonatal mortality. Overall survival was meticulously documented in medical records, culminating at the end of the study period in August 2022.

### Statistical analysis

The statistical analysis was conducted using SPSS Statistics for Windows (IBM SPSS Statistics for Windows, version 24.0. Armonk, NY: IBM Corp). The normality of the distribution was assessed using the Kolmogorov-Smirnov test. Quantitative variables were presented as mean ± standard deviation (SD) or median with interquartile range (IQR), depending on the normality of the data. Frequency and percentage values (n, %) were provided for qualitative variables. Comparative analyses were performed on the clinical data of patients with CoA and IAA. Furthermore, all patients were stratified based on neonatal mortality, and a comparative analysis of their data was conducted.

The chi-square test was used for analyses of qualitative variables. To compare two independent groups, the Student’s t-test was employed for variables demonstrating a normal distribution, whereas the Mann-Whitney U test was utilized for variables that did not conform to a normal distribution. Multiple regression analysis was executed to identify factors affecting mortality. Variables significant in pairwise comparisons (mode of delivery, age at diagnosis, age at first surgery, preoperative inotropic support, need for mechanical ventilation, STAT category, presence of major postoperative complications) were entered into the model. Each predictor variable was assessed using the Odds Ratio (OR) and 95% Confidence Interval (CI). Statistical significance was defined as p < 0.05.

## Results

The detailed echocardiographic data of 159 study patients are shown in Figure 1 and Central Illustration. Some patients had more than one lesion.

The demographic and clinical characteristics of the patients are presented in Table 1. Most patients (n=139, 87.4%) were transferred to our Cardiac NICU from other NICUs in Turkey. A total of 61 patients (38.3%) could have a correct diagnosis of CHD after discharge from NICUs of external centers.

**Table 1:** – The demographic and clinical characteristics of all study patients

Variables	N=159	CoA (N=120)	IAA (N=39)	p-value
Gestational age, week (mean±SD)	37.7±2.2	37.7±2.3	37.8±2.06	0.69
Birth weight, g (mean±SD)	2920±586	2939±593	2863±565	0.48
Male gender, n(%)	78 (49.1)	60 (50.0)	18 (46.2)	0.67
Delivery type, C/S, n (%)	100 (62.9)	77 (64.2)	23 (59.0)	0.56
Prematurity (<37 wk), n(%)	29 (18.2)	24 (20.0)	5 (12.8)	0.31
Age at diagnosis, day, median (IQR)	3 (1-7)	4 (1-7)	2 (1-5)	0.04
Age at admission, day, median (IQR)	6.0 (3-10)	6 (3-9)	6 (2-12)	0.95
Prenatal diagnosis, n(%)	29 (18.2)	19 (15.8)	10 (25.6)	0.16
**At preoperative period**				
Mechanical ventilation, n(%)	103 (64.8)	72 (60.0)	31 (79.5)	0.02
Inotropic support, n(%)	113 (71.1)	81 (67.5)	32 (82.1)	0.08
Sepsis, n(%)	61 (38.4)	40 (33.3)	21 (53.8)	0.02
Acute kidney injury, n(%)	34 (21.4)	25 (20.8)	9 (23.1)	0.76
Necrotizing enterocolitis, n(%)	7 (4.4)	6 (5.0)	1 (2.6)	0.51
**Cardiac catheterization, n(%)**				
*1st angiography*				
*Diagnostic*	42 (26.4)	25 (20.8)	17 (43.6)	
*Therapeutic*	51 (32.1)	49 (40.8)	2 (5.1)	0.002
*2st angiography*				
*Diagnostic*	5 (3.1)	2 (1.6)	3 (7.6)	
*Therapeutic*	12 (7.5)	11 (9.1)	1 (2.5)	0.02
**Cardiac Surgery, n(%)**				
**1st Surgery**	**135 (84.9)**	101 (84.1)	34 (87.1)	
***Thoracotomy***	112 (83.0)	96 (95.0)	16 (47.1)	
***Sternotomy***	23 (17.0)	5 (5.0)	18 (52.9)	<0.001
*CPB*	15 (11.1)	3 (3.0)	12 (35.3)	<0.001
*ECMO*	2 (1.5)	0 (0)	2 (5.9)	0.14
**2nd Surgery**	**36 (22.6)**	23 (19.1)	13 (33.3)	
***Thoracotomy***	17 (47.2)	13 (56.5)	4 (30.8)	
***Sternotomy***	19 (52.8)	10 (43.5)	9 (69.2)	0.13
*CPB*	14 (38.9)	6 (26.1)	8 (61.5)	0.03
*ECMO*	4 (11.1)	1 (4.3)	3 (823.1)	0.08
Life-threatening complications, n(%)	52 (38.8)	29 (29.0)	23 (67.6)	<0.001
NICU stay, day median (IQR)	24 (13-43)	23 (13-43)	24 (13-57)	0.09
Follow-up time, median (yr) (IQR)	1yr (2 month-6 yr)	3yr (2 month-7 yr)	2 month (1 month-2 yr)	0.001
Neonatal mortality, n(%)	**31 (19.5)**	16 (13.3)	15 (38.5)	0.001

CPB: Cardiopulmonary bypass; ECMO: Extracorporeal membrane oxygenation; NICU: Neonatal intensive care unit.

Fifteen (9.4%) patients were immigrants (Syrian or Iraqi). The preterm birth rate was 18.2% (n=29). In the preoperative period, all patients received PGE1 infusion. Twenty patients had a syndromic appearance: DiGeorge syndrome (n=6), Down syndrome (n=5), Turner syndrome (n=2), and trisomy 18 (Edwards) (n=1). Maternal Medical history was significant for gestational diabetes (n=3) and hypothyroidism (n=2). Hormone therapy was required for hypothyroidism in six patients.

In the neonatal period, cardiac catheterization was performed in 93 of 159 patients (58.4%).

Thrombosis (n=16, 17.2%), hypothermia (n=9, 9.6%), and bleeding (n=4, 4.3%) were the most common complications observed after catheterization. Of 135 patients undergoing surgery, life-threatening serious complications were noted in 38.5% (n=52).

The median length of NICU stay was 24 days (13-43). Neonatal mortalities (19.5%, n=31) were attributed to distinct factors, encompassing preoperative heart failure and sepsis (n=13), intraoperative arrest (n=8), and postoperative complications like sepsis, pneumonia, MODS, and/or hemodynamic instability (n=10).

### Coarctation of Aorta

During the neonatal period, at a median age of 11 days (6-28), 74 (61.6%) out of 120 patients with CoA underwent cardiac catheterization via the femoral artery and/or vein. Among them, 49 (66.2%) underwent therapeutic catheterizations (45 balloon angioplasty, four balloon valvuloplasty), while 25 (33.7%) underwent diagnostic catheterizations (see Table 1). The pressure gradient showed a significant decrease after angioplasty [30 (20-40) mmHg vs. 10 (5-14) mmHg, p<0.001)].

Following the initial angioplasty, 28 patients proceeded to surgery, and three required a second balloon angioplasty due to recoarctation, resulting in a recoarctation rate of 63.2% (n=31/49). Post-angioplasty, paradoxical hypertension requiring treatment was observed in eight of 45 patients (16.3%).

At a median age of 14 days (9.2-23 days), 101 patients with CoA (84.1%) underwent surgery (96-thoracotomy and 5-sternotomy). Nineteen of 120 patients (15.8%) did not undergo surgery; eleven received balloon angioplasty, and eight passed away before surgery.

Among 101 patients undergoing surgery, extended resection end-to-end anastomosis (n=66, 65.3%), resection end-to-end anastomosis (n=19, 18.8 %), and graft repair (n=16, 15.8%) were the surgical procedures. Three patients (2.9%) operated under CPB and moderate hypothermic circulatory arrest. Following surgery, paradoxical hypertension developed in 18 of 101 (17.8%) patients. The other frequent complications were sepsis, MODS, acute renal failure, chylothorax, and diaphragm paralysis. Three patients died during surgery, and five patients died due to postoperative sudden cardiac arrest or MODS.

At a median age of 88 days (48-350), 13 patients with CoA underwent the second catheterization; seven underwent balloon angioplasty, two diagnostic catheterizations, one atrial septostomy, and one VSD closure with Amplatzer Duct Occluder II (ADOII) device.

In infancy, 23 patients underwent second surgery with a recoarctation rate of 22.7% at a median age of 148 days (68-330). At this point, surgeries were performed via thoracotomy in 13 (56.5%) patients and median sternotomy in 10 (43.5%) patients. The types of surgeries were extended resection end-to-end anastomosis (n=16), graft repair (n=2), VSD patch (n=2), and LVOTO resection (n=3). Six of the patients underwent CPB.

### Interruption of the aortic arch

Of 39 patients, diagnostic catheterizations were performed in 17 (43.6%) at a median age of 7.5 days (3-15). Five patients with IAA (12.8%) were unable to have surgery because they passed away before surgery. Thus, 34 patients (87.1%) underwent IAA repair at a median age of 14 days (5-20) in the neonatal period.

Of 34 patients, 16 (47.1%) were operated on with thoracotomy and 18 (52.9%) with sternotomy; 12 (35.3%) underwent CPB. A single-stage repair was performed in 12 patients: two APW and aortic arch repairs with graft, nine end-to-end anastomoses, and VSD patch repair. Double-stage repair was implemented in 22 patients: 10 underwent aortic arch repair with graft and pulmonary banding, nine underwent end-to-end anastomosis with pulmonary banding, and three underwent end-to-end anastomosis without pulmonary banding. In the early postoperative period, two patients (5.9%) needed ECMO. The types of repair and median sternotomy rates were similar among patients with IAA type A and IAA type B (p >0.05 for both comparisons).

In infancy, at a median of 150 days (45-202), three patients required diagnostic catheterization, and one underwent therapeutic catheterization (endovascular stenting for para-anastomotic stenosis).

A total of 13 patients underwent second surgery at a median age of 177 days (56-330). At this step, surgeries were performed via thoracotomy in four patients and sternotomy in nine patients. Types of surgeries were six VSD patch repairs, four graft changes, and three pulmonary re-banding. Eight of the patients entered CPB, while three patients needed ECMO.

The most common postoperative complications were LCOS, sepsis/MODS, pneumonia, acute kidney injury, and arrhythmia. LCOS was defined in five patients with IAA and is closely related to preoperative left ventricular dysfunction. Two patients passed away during surgery, and eight died due to postoperative LCOS or MODS. The mortality rate was lower by double-stage repair, although not significantly (50.0% vs 34.8%, respectively, p=0.38).

### Outcomes

Central Illustration and Table 2 display the clinical features of the study patients according to neonatal mortality.

**Table 2:** – Clinical characteristics of the study patients according to the neonatal mortality

Variables	Survived (n=128)	Died (n=31)	p-value
Gestational age, week (mean±SD)	37.8±2.2	37.5±2.3	0.48
Birth weight, g (mean±SD)	2945±580	2818±609	0.28
Male gender, n(%)	64 (50.0)	14 (45.2)	0.62
Delivery type, C/S, n (%)	86 (67.2)	14 (45.2)	0.02
Prematurity, n(%)	22 (17.2)	7 (22.6)	0.48
Early diagnosis, n(%) (<3 day of life)	60 (46.9)	23 (74.2)	0.006
Age at 1st Surgery, day, median (IQR)	16 (10-25)	12 (5-15)	0.004
Prenatal diagnosis, n(%)	20 (15.6)	9 (29.0)	0.08
NICU stay (day), median (IQR)	28 (17-49)	5 (3-15)	<0.001
Mechanical ventilation, n(%)	76 (59.4)	27 (87.1)	0.004
Inotropic support, n(%)	86 (67.2)	27 (87.1)	0.02
Sepsis, n(%)	51 (39.8)	10 (32.3)	0.43
MODS, n(%)	23 (18.0)	11 (35.5)	0.03
Acute kidney injury, n(%)	25 (19.5)	9 (29.0)	0.24
Peritoneal dialysis, n(%)	6 (4.7)	3 (9.7)	0.28
Intracranial hemorrhage/hypoxic-ischemic changes, n(%)	19 (14.8)	3 (9.7)	0.45
Cardiac Surgery, n(%)			
CPB, n(%)	8 (7.0)	7 (33.3)	<0.001
ECMO, n(%)	0 (0)	2 (9.5)	0.001
Risk of surgery, median (IQR) STAT category	2 (2-4)	4 (4-4)	<0.001
Life-threatening complications, n(%)	34 (30.1)	18 (85.7)	<0.001

CoA: Coarctation of the aorta, Interrupted aortic arch; MODS: Multi-organ dysfunction syndrome; NICU: Neonatal intensive care unit; CPB: Cardiopulmonary bypass; ECMO: Extracorporeal membrane oxygenation; STAT: The Society of Thoracic Surgeons-European Association for Cardio-Thoracic Surgery.

In multivariate analysis, the higher STAT categories (OR:2.3, CI:95%, 1.1-5.1, p=0.03) and the presence of major postoperative complications (OR:9.8, CI:95%, 2.1-35.1, p=0.003) have increased the risk of neonatal mortality. The other variables did not affect the risk of mortality.

The median follow-up time of all patients was one year (2 months-6 years). Overall survival at the end of the study period was 71.0% (n=113/159).

## Discussion

The clinical presentations of CoA vary widely, encompassing infants who present with heart failure to asymptomatic individuals who exhibit systemic hypertension or murmurs detected incidentally during routine physical examinations in children and adults.^10,23^ CoA can also be associated with specific genetic syndromes, including Down, Turner, or Williams-Beuren Syndrome.^24,25^ In our study, the most prevalent presenting symptoms were cardiac murmurs, cyanosis, respiratory issues, and hemodynamic impairment. Some patients with CoA were associated with Down, Edwards, and Turner syndromes.

The management of complex lesions involving arch hypoplasia can be approached through a thoracotomy with an extended end-to-end anastomosis, aiming to enhance proximal arch growth by eliminating the distal obstruction.^26^ An alternative method involves midline sternotomy and enlargement of the aortic arch to achieve optimal relief of any obstruction in the left ventricular outlet.^27^ Dharmapuram et al.^28^ reported positive outcomes using extended end-to-end anastomosis through a thoracotomy with a modified surgical technique in CoA cases associated with proximal arch hypoplasia. Gropler et al.^29^suggested that median sternotomy should be considered for patients with severe arch hypoplasia. Graft patch aortoplasty is not recommended in infancy due to the associated risk of aneurysmatic dilatation.^30^

In our study, a total of 101 patients with CoA underwent surgery, and most of the cases were treated with thoracotomy. The preferred procedure for the majority of CoA patients was resection with extended end-to-end anastomosis. Approximately one-third of the CoA patients are associated with aortic arch hypoplasia. For this subgroup, the chosen surgical approach involved sternotomy with extended end-to-end anastomosis or the application of aortic patches. Graft repair was selectively performed in patients with long-segment hypoplasia. Notably, our team has not employed this specific surgical technique for Coarctation of the Aorta (CoA) repair in infants over the last five years.

Mid-to-long-term results highlight that recurrent aortic arch obstruction postoperatively remains a significant concern. In the study by Onalan et al*.,*^31^ it was found that 20.8% of patients required reintervention due to distal recoarctation, with seven successfully treated via aortic balloon angioplasty and three requiring surgical reintervention.

Following CoA repair, the persistence or recurrence of hypertension can be linked to factors such as later age at repair, the presence of residual obstruction, suboptimal aortic arch shape, and unclear neuro-humoral mechanisms initiated before the repair. The reported incidence of hypertension after CoA repair varies between 17 and 48% in numerous studies.^29-33^In our study, the rate of hypertension after coarctation surgery was 17.8%.

Currently, balloon angioplasty is the preferred approach for selected cases of CoA. In the neonatal period, angioplasty can be used as the first option to gain time until surgery, especially in cases of low birth weight and hemodynamic instability. In our study, approximately 40% of cases with CoA underwent angioplasty. In a study by Oswal et al. involving 44 infants, the mean gradient decreased from 48.05±15.26 mmHg to 10.97±5.8 mmHg, demonstrating successful immediate results after balloon angioplasty for CoA.^34^In the present study, all infants exhibited a peak gradient of more than 20 mmHg before intervention. Following angioplasty, there was a significant reduction in the pressure gradient [30 (20-40) mmHg vs. 10 (5-14) mmHg].

An IAA is a rare and severe CHD, which frequently coexists with other intracardiac and extracardiac defects.^8^ In our study, eight of the patients with IAA had associated complex heart defects such as APW, TGA, DORV, and tricuspid atresia. Mortality rates were similar among complex and isolated IAA types. Dealing with complex malformations poses surgical challenges, requiring intricate procedures. In such cases, palliation may be ineffective, and early complete repair is often necessary after birth.

In recent years, there has been a global trend toward promoting single-stage repair for IAA, but it requires complex technical skills and multidisciplinary cooperation.^12,13^Our study involving 39 IAA patients showed that 12 underwent single-stage repair, while 22 required double-stage repair.

In the neonatal period, postoperative serious complications like LCOS may occur after aortic arch repair, particularly when associated with preoperative left ventricular dysfunction. A study by Lim et al.^35^ reported a 5.7% postoperative LCOS rate in neonates or infants undergoing single-stage total repair of aortic arch anomalies with poor preoperative conditions. Our study also observed LCOS in five patients with IAA after surgery, all with preoperative left ventricular dysfunction. The presence of preoperative sepsis may have negatively impacted their outcomes.

Although both CoA and IAA are aortic arch pathologies,^1,2^ the prognosis for IAA is more severe.^7^ When comparing the two patient groups, it was noted that IAA cases were diagnosed earlier than CoA cases, possibly due to the more prominent symptoms. Similarly, the need for mechanical ventilation, the rate of sepsis, and the frequency of AKI were higher in IAA cases. In the presence of isolated CoA, angioplasty can provide a temporary solution, whereas in IAA, angiography is used for diagnostic purposes, with treatment being solely surgical. Therefore, in our study, the frequency of therapeutic angiography was higher in CoA cases, while diagnostic angiography was more frequent in IAA cases.

While surgeries for isolated CoA can be performed using minimally invasive methods and thoracotomy, IAA procedures are frequently carried out under CPB and through sternotomy. Consequently, the frequency of sternotomy was higher among IAA cases. Besides, as previously reported,^8^ due to the complexity of the surgery, the STAT category, ECMO requirement, and mortality rates were found to be higher in IAA cases.

When comparing surviving and deceased cases, it was observed that those who died received an earlier diagnosis. This phenomenon was attributed to the severity of the disease, leading to earlier symptom manifestation in babies. Additionally, due to the earlier surgical intervention required for more severe cases, the age at surgery for deceased patients was lower, and their STAT categories were higher. Compared to survivors, deceased patients had a higher need for preoperative mechanical ventilation and inotropic support, and the rate of MODS was more frequent. In the deceased group, the requirements for CPB and ECMO, as well as the frequency of life-threatening postoperative complications, were higher. In multivariate regression analysis, the factors most significantly impacting mortality were the STAT category, which reflects the complexity of the surgery and the development of severe postoperative complications.

This study has limitations, including its retrospective and single-center design, hindering the generalizability of results. Multi-center studies with long-term outcomes are necessary for a comprehensive understanding of congenital aortic arch anomalies.

In conclusion, newborns with congenital duct-dependent aortic anomalies require optimal perioperative care due to their increased risk of morbidity and mortality. In developing countries, improving neonatal CHD outcomes seems possible via several factors, including increased knowledge and expertise of physicians, the reached learning curve, adaptation to medical and technical developments, and keeping up advances in neonatal cardiac surgery.
